# Intravenous immunoglobulin for patients with unexplained recurrent implantation failure: a 6-year single center retrospective review of clinical outcomes

**DOI:** 10.1038/s41598-024-54423-z

**Published:** 2024-02-16

**Authors:** Einav Kadour Peero, Shorooq Banjar, Rabea Khoudja, Shaonie Ton-leclerc, Coralie Beauchamp, Joanne Benoit, Marc Beltempo, Michael H. Dahan, Phil Gold, Isaac Jacques Kadoch, Wael Jamal, Carl Laskin, Neal Mahutte, Simon Phillips, Camille Sylvestre, Shauna Reinblatt, Bruce D. Mazer, William Buckett, Genevieve Genest

**Affiliations:** 1 Department of Obstetrics and Gynecology, Division of Reproductive Endocrinology and Infertility, Bnai-Zion Medical Center, Faculty of Medicine, Technion - Israel Institute of Technology, Haifa, Israel; 2 Division of Clinical Immunology and Allergy, Department of Internal Medicine, King Abdulaziz University, Jeddah, Saudi Arabia; 3 Division of Clinical Immunology and Allergy, Department of Medicine, McGill University Health Centre, Montréal, QC Canada; 4 Department of Medicine, Queen’s University, Kingston, ON Canada; 5 Ovo Clinic, 8000 Boulevard Decarie, Montréal, QC H4P 2S4 Canada; 6 Obstetrics and Gynaecology Department, University of Montreal, 2900 Edouard Montpetit Blvd, Montreal, QC H3T 1J4 Canada; 7 Division of Neonatology, Montreal Children’s Hospital – McGill University Health Centre, Montreal, QC Canada; 8 Division of Reproductive Endocrinology and Infertility, Department of Obstetrics and Gynecology, McGill University, McGill University Health Centre, Montréal, QC Canada; 9 Department of Allergy and Immunology, Montreal General Hospital, 1650 Cedar Ave. A6-123, Montreal, QC H3G 1A4 Canada; 10 Clinique OVO, 8000 boulevard Décarie, Montréal, QC H4P 2S4 Canada; 11 Department of Obstetrics and Gynecology, Division of Reproductive Endocrinology and Infertility, Faculty of Medicine, University of Montreal, Montreal, QC Canada; 12 TRIO Fertility, 655 Bay St, Toronto, ON M5G 2K4 Canada; 13 Deptartments of Medicine and Obstetrics & Gynecology University of Toronto, 27 King’s College Cir, Toronto, ON M5S Canada; 14 The Montreal Fertility Centre, 5252 de Maisonneuve Blvd West, Suite 220, Montreal, QC H4A 3S5 Canada; 15 Faculty of Medicine, University of Montreal, 2900 Edouard Montpetit Blvd, Montreal, QC H3T 1J4 Canada; 16 Division of Reproductive Endocrinology and Infertility, University of Montreal, 2900 Edouard Montpetit Blvd, Montreal, QC H3T1J4 Canada; 17 McGill University Health Care Reproductive Center, 888 Boul. De Maisonneuve E# 200, Montreal, QC H2L 4S8 Canada; 18 Department of Pediatrics, Division of Allergy Immunology and Clinical Dermatology, Montreal Children’s Hospital, McGill University, Montréal, QC Canada; 19 Program in Translational Research in Respiratory Diseases, Research Institute of the McGill, University Health Centre, Montréal, QC Canada

**Keywords:** Unexplained recurrent implantation failure, Intravenous immunoglobulin, Reproductive immunology, Immune-mediated recurrent implantation failure, Immunology, Medical research

## Abstract

The effectiveness of intravenous immunoglobulin (IVIg) for patients with unexplained recurrent implantation failure (uRIF) remains debated. We retrospectively analysed outcomes of uRIF patients treated with IVIg compared to a separate control uRIF cohort within our center (01/2014–12/2021). Primary outcomes included live birth, miscarriage, or transfer failure. We documented IVIg side effects and maternal/fetal outcomes. Logistic regression analysis was used to assess for association of IVIg exposure with outcomes and adjust for confounders. The study included 143 patients, with a 2:1 ratio of controls to patients receiving IVIg treatment. Patient characteristics were similar between groups. There was higher live birth rate (LBR) in patients receiving IVIg (32/49; 65.3%) compared to controls (32/94; 34%); p < 0.001). When stratifying patients into moderate and severe uRIF (respectively 3–4 and $\ge$ 5 previous good quality blastocyst transfer failures), only patients with severe uRIF benefited from IVIg (LBR (20/29 (69%) versus 5/25 (20%) for controls, p = 0.0004). In the logistic regression analysis, IVIg was associated with higher odds of live birth (OR 3.64; 95% CI 1.78–7.67; p = 0.0004). There were no serious adverse events with IVIg. IVIg can be considered in well selected patients with $\ge$ 5 previous unexplained, high quality blastocyst transfer failures. A randomized controlled trial is needed to confirm these findings.

## Introduction

In Canada, infertility affects 1/6 couples (2022 CARTR report). While in-vitro fertilization (IVF) has revolutionized the treatment of infertility, up to 5% of IVF patients will experience recurrent implantation failure (RIF), generally defined as absence of intrauterine pregnancy after $\ge$ 3 high quality single blastocyst transfers^1^. Over 50% of RIF couples will receive a diagnosis of unexplained RIF (uRIF) despite extensive investigation of both male and female partners^2,3^. Unexplained RIF represents a vulnerable and understudied patient population. For couples with RIF, the prognosis for live birth is lower than IVF patients without RIF, with historical RIF cohorts estimating LBR of 12–20% per embryo transferred^4^. The financial, physical, and emotional toll on affected couples is further compounded by the paucity of therapies or interventions available to improve pregnancy rates^2^. While the most common cause of RIF is embryo aneuploidy^3,5^, up to 7% of women transferring euploid embryos fail to have a live birth after three cycles of single blastocyst transfer^6^, suggesting other unknown causes.

An immune contribution to RIF has gained much attention in the last decade. Indeed, the cycling endometrium is characterized by extensive immune remodeling during endometrial regeneration and decidualization, with endometrial immune cells (natural killer (NK) cells, macrophages, dendritic cells and T cells) progressively acquiring specialized functions required for optimal endometrial receptivity, spiral artery remodeling, embryo recognition; and to guide trophoblast invasion and tolerance^7^. Thus, a dysfunctional endometrial immune response may explain a proportion of uRIF cases. Unfortunately, no biomarkers exist to confirm a diagnosis of immune-mediated RIF (IM-RIF). While peripheral blood testing including NK cell enumeration and function, T helper cell 1 (Th1) to T helper cell 2 (Th2) ratios as well as pro-inflammatory to anti-inflammatory cytokine ratios are used in some clinics, these tests lack validation^8^, do not correlate with reproductive outcomes^9,10^, and do not seem to reflect the endometrial immune micro-environment^11^. Endometrial immunophenotyping tests (Ultimpro®) are available and may represent a better diagnostic and prognostic tool^12,13^, but they have not been validated by an independent third party.

Currently, IM-RIF is a diagnosis of exclusion and is clinically suspected in otherwise good prognosis patients with uRIF, especially in patients having failed multiple euploid blastocyst transfers^2^. A large number of treatments aiming to modulate the immune system have been trialed, none having convincingly or uniformly improved IVF outcomes^7^. Among such treatments, intravenous immunoglobulin (IVIg) has been studied as an adjunct therapy for almost 30 years. IVIg is a plasma-derived product that contains polyclonal immunoglobulin G and is used in clinic to treat patients with humoral immune deficiency and autoimmune diseases^14^. It is safe and well tolerated in pregnancy, even at high doses^15,16^. As an immune modulator, the effects of IVIg are multipronged^17^, reducing both innate and adaptive immune responses while possibly improving immune tolerance required for successful implantation. However, being a widely used blood product, IVIg is subject to high production costs and shortages^18^ and must be used judiciously to ensure proper resource management.

While most recent systematic reviews and meta-analysis on IVIg efficacy for RIF suggest a positive effect^19–22^, the available literature is very heterogenous and difficult to interpret. Definitions of RIF are not standardized, and key confounding variables are often not detailed (e.g. embryo quality, embryo or blastocyst transfer, embryo ploidy and endometrial preparation). IVIg dose, brand, timing of administration as well as co-treatment with other immune modulators vary widely, and available randomized controlled trials (RCT) are often underpowered to detect treatment effects^23–32^. Data amassed thus far is insufficient to recommend routine IVIg use for RIF and patients most likely to benefit from IVIg remain undefined. In this study, we present a retrospective review of our clinic’s 6-year outcomes, comparing IVIg success rates to ‘expected outcomes’ from a separate control cohort.

## Methods

### Study design and setting

This is a retrospective cohort study of patients evaluated and followed at The McGill University Health Center (MUHC) Reproductive Immunology Clinic (MRIC) and the MUHC Reproductive Center. The MRIC is the reference center in the province of Quebec for the immunologic evaluation of patients with uRIF. The MUHC Reproductive Center is separate from the MRIC and is a MUHC affiliated fertility clinic. We aimed to determine if IVIg improved outcomes in patients with RIF. This study was approved by the MUHC research ethics board (MUHC REB #2022-8157). All methods were performed in accordance with the relevant guidelines and regulations. This manuscript was written according to Strengthening the Reporting of Observational studies in Epidemiology (STROBE) guidelines.

In Quebec IVIg is publicly funded but tightly regulated. All patients must be referred to and evaluated at the MRIC for IVIg eligibility. Criteria for IVIg are: $\ge$ 3 unexplained high-quality blastocyst transfer failures, age < 42 (< 45 if using oocyte donation), body mass index (BMI) < 35, non-smokers, and failure of previous medical therapy for RIF (switching ET protocols, endometrial scratch, low dose aspirin). Criteria for a high-quality blastocyst is $\ge$ 3BB (Gardner’s criteria) for transfers conducted after 2013 and ‘grade 1 or 2’, ‘expanded’ or ‘hatching’ blastocysts for transfers prior to 2013. Generally, all eligible patients consented to IVIg treatment. However, between January 2021 and December 2022, IVIg became unavailable for the treatment of RIF in Quebec due to COVID-imposed resource allocation.

### IVIg protocol

Privigen® (0.6–0.8 g/kg of ideal body weight) was administered as a slow infusion 5–10 days prior to embryo transfer in a monitored outpatient hospital setting. This dose was repeated monthly until 16–20 weeks in patients who achieved pregnancy. For patients who experienced side effects with IVIg, the IVIg dose was either split over 2 days or patients were offered subcutaneous immunoglobulin administration (Hizentra® 0.2 g/kg weekly until 16–20 weeks) once pregnancy was diagnosed. Each patient was required to sign our clinic’s standard informed consent form for IVIg infusion and was extensively counseled on the risks of IVIg as well as its off-label use in uRIF. Prior to the first IVIg infusion, serologies (Parvovirus, Cytomegalovirus, Toxoplasma, Rubella, Varicella), a complete blood cell count, a creatinine level and liver enzymes were obtained. IgA levels are not measured as there is no clear association between IgA deficiency and IVIg-induced anaphylaxis^33^.

### Study population

#### Intervention group

All patient with uRIF receiving IVIg treatment between January 1st 2014 to December 31st 2020 at the MRIC were included (See inclusion/exclusion criteria, Table 1). The first embryo transfer (ET) treated with IVIg was used as the index ET. Patients were excluded from the IVIg group if there was a significant loss of embryo quality upon thaw. Patients resorting to third party reproduction (oocyte donation) were included if they had failed $\ge$ 3 high quality oocyte donor blastocyst transfers. Patients with endocrine disease (diabetes, hypothyroidism), thrombophilia and persistently positive anti-phospholipid antibodies were included, provided their underlying condition was well controlled and they had failed at least 1 embryo transfer with medical correction or treatment of their condition.

**Table 1:** Inclusion/exclusion criteria for IVIg treatment.

Inclusion criteria	
Age	> 25 to < 42 if using own oocytes (< 45 if using oocyte donation)
BMI	< 35 kg/m^2^ or weight < 95 kg
Genetic	Normal parental karyotypes or euploid embryos
Endometrium	Trilaminar $\ge$ 7 mm at time of ET
Anatomic	Normal uterine cavity, absence of hydrosalpinges
Medical	No endocrinopathy, coagulopathy (or failure $\ge$ 1 ET with medical correction)*
Infectious	Absence of HIV, hepatitis B/C and syphilis
Other	Non-smoker
Exclusion criteria	
Medical	Current or previous history of: - Thrombosis** - Renal failure - Unprovoked cytopenia
Co-treatment with other immune modulators*	- Granulocyte colony stimulating factor (G-CSF) - Low molecular weight heparin (LMWH) - Corticosteroids - Calcineurin inhibitors
Other	Non-compliance with IVIg

Co-treatment with other immune modulators was allowed if the patient had previously failed an ET with the same treatment.

*Patients with endocrine disease were included provided their underlying condition was well controlled and they had failed at least 1 embryo transfer with medical correction of endocrinopathy. Patients with coagulopathy (hereditary thrombophilia or persistently positive anti-phospholipid antibodies) were included provided they had failed at least 1 embryo transfer with medical treatment (aspirin or low molecular weight heparin).

**Patients with coagulopathy and a history of unprovoked thrombosis were excluded.

#### Control group

We selected a separate unmatched ‘natural history’ cohort of patients with similar maternal characteristics and reproductive histories as the IVIg-treated patients. We aimed to include approximatively 2 controls per patient in the IVIg treated group. To achieve this estimated sample size, we included patients from both the MUHC Reproductive Center and the MRIC followed between January 2020 and December 2021.

Control patients from the MUHC Reproductive Center, were included if they had $\ge$ 3 previous high quality blastocyst transfer failures and respected the inclusion criteria (Table 1). The last blastocyst transfer on record was included as the index ET.

Control patients from the MRIC were included if they met criteria for IVIg but did not receive IVIg (either because of delays in embryo transfer or because of COVID-imposed IVIg treatment restrictions). For the MRIC control patients, the last ET on record during the study period (January 2020-December 2021) was included as the index ET.

Since patients with uRIF at the MUHC Reproductive clinic are often referred to the MRIC, we ensured there was no overlap between the natural history control cohort and the IVIg cohort. All patients in the control cohort received standard of care for their index ET. Of note, parental karyotyping was not part of the standard workup for RIF, control patients were included even if karyotyping was not performed. Similarly, BMI was not available for many control patients, but weight was available for all. We excluded patients over 95 kg (BMI 34.9 for an average 165 cm woman). Patients were excluded if they had previous access to other immunomodulatory treatments including glucocorticoids (with the exclusion of Medrol as this steroid is often featured as standard protocols in some Quebec IVF clinics), intralipids, tacrolimus or intravenous immunoglobulin at any time prior to inclusion in this study. Co-treatment with aspirin was not excluded as it is a frequent adjunct in standard IVF protocols and has not been shown to improve LBR in RIF^7^.

For both the intervention and control groups, only patients for whom the index embryo transfer outcome was known were included in the analysis. All data was collected via retrospective chart review using institutional databases.

### Variable definition

Primary RIF was defined as RIF in the absence of previous intra-uterine pregnancy. Secondary RIF was defined as $\ge$ 3 failed high quality blastocyst transfers after $\ge$ 1 intra-uterine pregnancy (whether this was a miscarriage or a live birth).

Primary outcomes included live birth, miscarriage or embryo transfer failure. Pregnancy was defined as the presence of an intra-uterine gestational sac; a successful outcome was defined as a live birth occurring $\ge$ 24 gestational weeks (GW); unsuccessful outcomes included implantation failure or miscarriage < 24 GW after ET. A biochemical pregnancy was defined as a positive quantitative hCG test without evidence of intra-uterine pregnancy.

Based on co-authors consensus, moderate RIF was defined as 3–4 good quality blastocyst transfers and severe RIF was defined as $\ge$ 5 good quality blastocyst transfers prior to index ET.

### Statistical analysis

We had initially planned to match case and control patients for age and RIF severity, however, we could not find enough control patients with $\ge$ 3 unexplained high quality blastocyst transfer failures. Indeed, most controls with RIF are eventually referred to the MRIC and offered immunomodulatory therapy. To compensate for lack of matching, we chose to include a 2:1 ratio of control to IVIg treated patients.

Patient were stratified into primary RIF (RIF-1) and secondary RIF (RIF-2) to preserve group homogeny; patients exposed to IVIg were compared to control patients within each strata. Continuous variables were presented as median and minimum–maximum values or mean and standard deviation; categorical data were presented as percentages. We used Shapiro Wilk tests to assess normal distribution of the quantitative parameters; we used the Mann Whitney *U* test (Student t-test for parity only) for continuous variables and the Fisher exact test for categorical data.

For each strata (RIF-1 and RIF-2), logistic regression analysis was conducted to evaluate the association of IVIg (vs no IVIg) with live birth and to adjust for potential confounders (maternal age at embryo transfer and number of previous failed transfers prior to index pregnancy).

To evaluate if the association of IVIg with live birth differed between the RIF-1 and RIF-2 groups, we combined both groups in an exploratory analysis. Logistic regression analysis was used to evaluate the association of IVIg with live birth and adjusted for type of RIF (RIF-1 or RIF-2), number of previously failed good quality blastocyst transfers and maternal age at the time of index transfer. An interaction factor was used to evaluate the interaction between IVIg and type of RIF (RIF-1 and RIF-2). A p-value for interaction of < 0.05 would indicate that the association of IVIg with live birth is statistically significantly different between the Primary and Secondary RIF groups. Analyses were performed using R version 3.6.0 (R Foundation for Statistical Computing, Vienna, Austria).

The authors have used appropriate statistical methods for analysis. Raw data is available on demand for editorial review.

### Ethics approval and consent to participate

This study was approved by the McGill University Health Center (MUHC) ethics board, study number MUHC REB # 2022-8157.

## Results

### IVIg cohort

**Figure 1:**
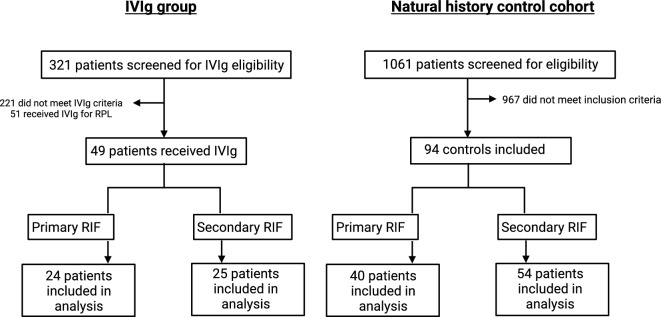
Patient enrollment and study design.

Between January 1st 2014 and December 31st 2020, a total of 321 patients with reproductive failure were assessed at the MRIC for IVIg eligibility. Forty nine patients received IVIg for RIF and 221 did not meet eligibility criteria. Twenty-four patients received IVIg for primary RIF (RIF-1) and 25 patients received IVIg for secondary RIF (RIF-2) (Fig. 1).

### Natural history (control) cohort

Between January 1st 2020 and December 31st 2021, 1061 patients were screened for the ‘natural history’ control group (891 patients from the MUHC Reproductive Center and 170 patients from the MRIC). Of these patients, 94 patients met inclusion criteria (Table 1) and were included as controls, 40 in the primary RIF (RIF-1) control group and 54 in the secondary RIF (RIF-2) control group (Fig. 1).

### Patient characteristics

Patient baseline characteristics are present in Table 2. Overall, IVIg and control groups were similar in terms of maternal age at index embryo transfer, anti-mullerian hormone (AMH) levels and factors contributing to infertility. In the RIF-1 group, patients receiving IVIg had a higher number of previous blastocyst transfer failures as well as a higher number of previous total embryo transfer failures (including lesser quality blastocysts, morula and day 3 embryos) compared to control. In the RIF-2 group, patients receiving IVIg had a higher total number of previous total embryos transfer failures.

**Table 2:** Baseline patient characteristics.

Variable	IVIg group (n = 24)	Control group (n = 40)	P-value
Primary RIF (RIF-1)			
Age at embryo transfer (years)	35.5 (26–44)	34.0 (24–41)	0.083
$\le$ 35, n (%)	12 (50)	28 (70)	
> 35, n (%)	12 (50)	12 (30)	
BMI (kg/m^2^)	22.0 (18.3–34)	23.3 (19–31.1)	0.25
< 25, n (%)	16 (66.6)	22 (55.0)	1
25–34.9, n (%)	8 (33.3)	12 (30.0)	
Missing data, n (%)	0	6 (15.0)	
# blastocyst transfers before index ET*	5.0 (4–9)	4.0 (3–6)	< 0.001***
3–4 blastocyst transfers, n (%)	7 (29.2)	32 (80)	
≥ 5 blastocyst transfers, n (%)	17 (70.8)	8 (20)	
Total # failed ET before index ET**	6.0 (4–17)	4.0 (3–9)	< 0.001***
AMH (ng/mL)	2.79 (0.12–8.24) (n = 19)	2.53 (0.66–10) (n = 28)	0.8
Reason for IVF (%)			
Unexplained	12 (50)	23 (57.5)	0.51
Explained (one patient may have several)	12 (50)	17 (42.5)	
Male factor, n (%)	10 (41.7)	22 (55.0)	
Tubal factor, n (%)	0	1 (2.5)	
Diminished ovarian reserve, n (%)	4 (16.7)	0	
Pre-implantation genetic screening, n (%)	5 (20)	5 (12.5)	
Egg donation, n (%)	2 (8.3)	0	
PCOS, n (%)	3 (12.5)	5 (12.5)	
Endometriosis, n (%)	2 (8.3)	6 (15)	

Variable	IVIg group (n = 25)	Control group (n = 54)	P-value
Secondary RIF (RIF-2)			
Age (years)	36.0 (32–42)	36.0 (26–44)	0.28
$\le$ 35, n (%)	10 (40)	25 (46.3)	0.65
> 35, n (%)	15 (60)	29 (53.8)	
BMI (kg/m^2^) (range)	22.0 (17.9–32)	23.0 (19–32.6)	0.89
< 25, n (%)	17 (68.0)	19 (35.2)	0.37
25–34.9, n (%)	8 (32.0)	13 (24.1)	
Missing data, n (%)	0	18 (33.3)	
Gestation prior to index pregnancy (number)	3.0 (1–5)	2.0 (1–5)	0.065
Parity prior to index pregnancy (mean, SD)	0.48 (+ / − 0.714)	0.63 (+ /– 0.560)	0.16
# of blastocyst transfers before index ET*	4.0 (3–8)	4.0 (3–10)	0.14
3–4 blastocyst transfers, n (%)	13 (52)	37 (68.5)	0.21
≥ 5 blastocyst transfers, n (%)	12 (48)	17 (31.5)	
Total # failed ET before index ET**	5.0 (3–12)	4.0 (3–13)	0.005***
AMH (ng/ml)	2.64 (0.65–10.4) (n = 18)	2.73 (0.56–16.5) (n = 31)	0.79
Reason for IVF (%)	–	–	–
Unexplained	16 (64)	40 (74.1)	0.43
Explained (one patient may have several)	9 (36)	14 (25.9)	
Male factor, n (%)	11 (44)	36 (66)	
Tubal factor, n (%)	2 (8)	10 (18.5)	
Diminished ovarian reserve, n (%)	3 (12)	1 (1.9)	
Pre-implantation genetic screening, n (%)	4 (16)	15 (27.8)	
Egg donation, n (%)	0	0	
PCOS, n (%)	1 (4)	15 (27.8)	
Endometriosis, n (%)	6 (24)	9 (16.7)	

All p-values were calculated using the Mann Whitney *U* test for continuous variables (expressed as median and (minimum–maximum value)) or the Fisher exact test or Student T-test for categorical variables (+ /– indicated mean and standard deviation).

*BMI* body mass index, *ET* embryo transfer, *PCOS* polycystic ovarian syndrome, *IVIg* intravenous immunoglobulin.

*Only high-quality blastocyst ($\ge$ 3BB).

**Total blastocysts (including day 3, morula and lesser quality blastocysts (< 3BB).

***Statistically significant.

### IVIg outcomes

Because the immune mechanism(s) of RIF and probability of live birth may differ between patients with primary and secondary RIF, we first analyzed each group (RIF-1 and RIF-2) separately to preserve group homogeneity. In the RIF-1 group, there was a higher live birth rate (LBR) with IVIg compared to controls (14/24 (58.3%) vs 8/40 (20%); p = 0.0027). For patients in the RIF-2 group, IVIg similarly improved LBR compared to controls (18/25 (72%) vs 24/54 (44%); p = 0.03) (Table 3). Upon logistic regression analysis, adjusting for maternal age at time of index embryo transfer, IVIg improved the odds of live birth compared to control in both RIF-1 and RIF-2 groups (RIF-1 OR: 6,78, 95% CI (2.13–24.35), p = 0.0018) and RIF-2 OR: 2.92, 95% CI (1.06–8.69), p = 0.043) (Supplementary Appendix).

**Table 3:** Cohort outcomes.

Primary RIF (RIF-1)	RIF-1 IVIg	RIF-2 control	P-value
Variable	IVIg Group n = 24	Control group n = 40	
Live birth (%)	14/24 (58.3)	8/40(20)	0.0027*
Live birth subgroup with < 5 failed blastocyst transfers (%)*	4/7 (57.14)	8/32 (25)	0.17
Live birth subgroup with ≥ 5 failed blastocyst transfers (%)*	10/17 (58.8)	0/8	0.007*

Secondary RIF (RIF-2)	RIF-2 IVIg	RIF-2 control	P-value
Variable	IVIg group n = 25	Control group n = 54	
Live birth (%)	18/25 (72.0)	24/54 (44.4)	0.03**
Live birth subgroup with < 5 failed blastocyst transfers (%)*	8/13 (61.5)	19/37 (48.6)	0.74
Live birth subgroup with ≥ 5 failed blastocyst transfers (%)*	10/12 (83.3)	5/17 (29.4)	0.008**

Primary and secondary RIF combined			
Variable	IVIg group n = 49	Control group n = 95	P-value
Live birth (%)	32/49 (65.3)	32/95 (34)	< 0.001**
Live birth subgroup with < 5 failed blastocyst transfers (%)*	12/20 (60)	27/69 (39.1)	0.126
Live birth subgroup with ≥ 5 failed blastocyst transfers (%)*	20/29 (69)	5/25 (20)	0.0004**

All p-values were calculated with Fisher exact test.

*Only high-quality blastocyst ($\ge$ 3BB).

**Statistically significant.

We then hypothesized that the probability of live birth would differ between patients moderate RIF (3–4 previously failed high quality blastocyst transfers) compared to those with severe RIF ($\ge$ 5 previously failed high quality blastocyst transfers). We stratified patients in both groups (RIF-1 and RIF-2) into sub-categories depending on RIF severity (moderate, severe) and analyzed each group (RIF-1, RIF-2) separately.

In the RIF-1 group, IVIg improved LBR for patients with severe RIF (10/17 (58.8%) IVIg vs 0/8 (0%) in controls; p = 0.007). For patients with moderate RIF, there was a trend towards improved live birth with IVIg, but this was not significant (4/7 (57.1%) IVIg vs 8/32 (25%) in controls; p = 0.17). Similarly, in the RIF-2 group, only patients with severe RIF benefited from IVIg (10/12 (83.3%) live birth with IVIg vs 5/17 (29.4%) in controls; p = 0.008); patients with moderate RIF-2 did not (8/13 (61.5%) live birth IVIg vs 19/37 (48.6%) controls; p = 0.74) (Table 3). Logistic regression analysis was performed for both groups (RIF-1 and RIF-2), adjusting for maternal age at time of index embryo transfer and number of previously failed embryo transfers. For patients with primary RIF (RIF-1), IVIg improved the odds of live birth compared to control (OR 10.14, 95% CI (2.44–52.13), p = 0.0026); similar results were found for patients with secondary RIF (RIF-2) (OR 2.94, 95% CI (1.06–8.82), p = 0.043) (Supplementary Appendix).

Lastly, we sought to determine if the *effect* of IVIg was different depending on the type of RIF (RIF-1 or RIF-2). If the effect of IVIg similarly improves LBR for patients with RIF-1 and RIF-2, both groups do not need to be distinguished, facilitating patient recruitment for future RCT studies. An interaction factor was used to evaluate the interaction between IVIg and type of RIF (RIF-1 and RIF-2). The p-value for the interaction of IVIg and type of RIF was 0.41 (not significant) (Supplementary Appendix), meaning that the association of IVIg with live birth is similar between RIF-1 and RIF-2 groups; both groups can be combined for analysis.

We first combined both primary (RIF-1) and secondary (RIF-2) groups, finding as expected, that the LBR was higher amongst IVIg treated patients compared to control (32/49 (65.3%) vs 32/94 (34.0%); p < 0.001). Again, we stratified patients depending upon RIF severity, finding that only patients with severe RIF benefitted from IVIg (LBR with IVIg 20/29 (69%) versus 5/35 (20%) controls, p = 0.0004); there was no significant benefit for patients with moderate RIF (LBR with IVIg 12/20 (60%) vs 32/95 (34%), p = 0.126 (Table 3). Then, we performed logistic regression of the whole study group, adjusting for age at index embryo transfer and number of previously failed embryo transfers, showing a beneficial effect of IVIg on live birth (OR 3.63, 95% CI (1.69–8.05); p = 0.0011) (Supplementary Appendix).

Overall, pregnancy rates in the IVIg treated group were 35/49 (69.4%) and in the control group were 39/95 (41%) (p = 0.057). There were 3 pregnancy losses (3/49, 6.12%) (all biochemical pregnancies) in the IVIg group compared to 7 (7/94, 7.45%) pregnancy losses in the control group (4 biochemical pregnancies, 3 early (< 6 weeks) clinical pregnancy losses), without statistical significance (p = 1).

### IVIg safety

IVIg was generally well tolerated. Seven patients (7/49 (14.3%)) reported adverse events with immunoglobulin treatment. Three patients reported moderate headache (6.1%) and 2 (4.1%) patients reported cutaneous symptoms (urticaria and nummular eczema) post IVIg infusion. One patient received split IVIg dosing and completed the treatment protocol; two patients received Hizentra, both reporting mild local infusion reactions (local swelling, pain, and redness) and completed treatment. There were no cases of anaphylaxis, infusion reaction, aseptic meningitis, acute viral infection, or hemolytic anemia.

### Maternal and neonatal complications

There were no reported adverse maternal, obstetrical, or neonatal outcomes in the RIF-1 control group. In the RIF-1 IVIg group, one patient with autoimmune polyendocrinopathy was diagnosed with pre-eclampsia at 37 weeks and was induced to deliver a healthy 3265 g daughter. In the RIF-2 control group, there were 2 adverse events. One patient was diagnosed with gestational diabetes mellitus (GDM); another delivered a healthy 1880 g male prematurely at 32 weeks due to pre-term premature rupture of membranes. In the RIF-2 IVIg group, there were 7 reported adverse events. Two patients developed GDM (neither had received glucocorticosteroids). One patient had placenta accreta requiring term C-section of a healthy 4407 g boy. One patient had labor induced at 36 weeks due to cholestasis of pregnancy; her infant did not require hospitalization and had a normal birth weight (2767 g). Another patient developed severe pre-eclampsia with pre-term delivery at 32 weeks (healthy 1640 g female). Two patients developed post-partum hemorrhage not requiring blood transfusions. Of note, for patients having normal term deliveries, we did not systematically record neonatal birthweight. Most patients in the control groups delivered in other hospitals, data collection may be incomplete regarding obstetric and neonatal complications.

## Discussion

Intravenous immunoglobulin has been used for almost 30 years to treat patients with uRIF. The exact mechanism by which it may improve reproductive outcomes remains misunderstood; and there are currently no widely accepted guidelines to determine eligibility for IVIg^34^. Patient selection for IVIg treatment has historically been based on exclusion of alternative diagnoses for RIF, previous treatment failure or variances in peripheral blood immune testing. Thus, IVIg remains a hotly debated IVF adjunct therapy. Efforts have been made to synthesize the literature^20,35^, but meta-analysis of available studies have been limited by significant heterogeneity. Finally, true RIF remains a rare diagnosis^5^. While a well powered RCT is needed to evaluate the effect of IVIg for women with RIF, repeating an RCT without properly selecting eligible patients would likely yield similar results to the ones already published. Therefore, we appraised the outcomes of our past 6 years of clinical utilization of IVIg to determine (1) ideal candidates for IVIg, (2) ideal timing of IVIg administration and (3) ideal IVIg dosing prior to designing a protocol for an RCT.

Our criteria for IVIg administration (Table 1) included young patients with unexplained high quality blastocyst transfer failures, a normal endometrium at time of transfer with a BMI < 35 and non-smoking. By stringently selecting our study population and excluding confounding variables that may decrease implantation success, we were hoping to enrich our IVIg cohort with patients that have a true diagnosis of immune-mediated RIF. Our control population, like other studies^24,25,28,30,32,36^ included patients with similar maternal characteristics and reproductive histories as the IVIg-treated patients. The LBR for our control population is comparable to what has been previously published (LBR 12–35% after a diagnosis of RIF^4,37^).

**Figure 2:**
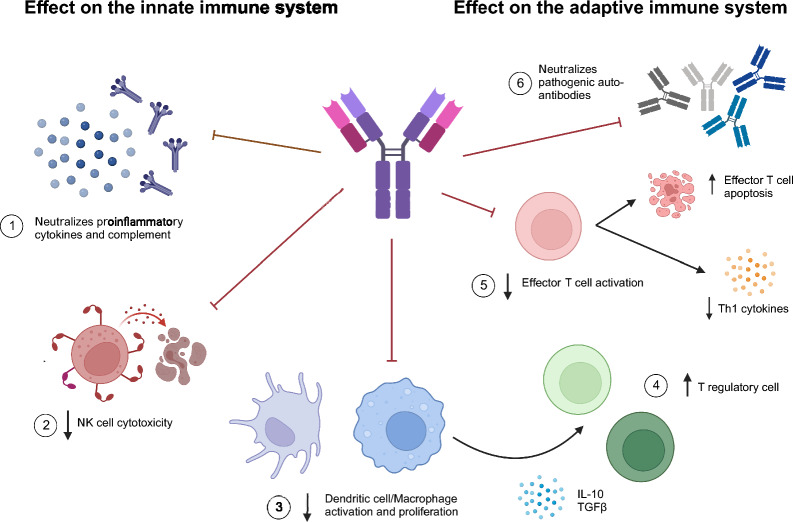
IVIg mechanisms of action. In murine studies, intravenous administration of IgG has been shown to penetrate into the uterus and exert local effects in addition to systemic ones^49^. IVIg can neutralize pro-inflammatory cytokines, chemokines and complement components, reducing the overall inflammatory milieu (**1**). IVIg has a direct impact on natural killer (NK cells), reducing their activation status, and cytotoxic potential (**2**). IVIg has been shown to profoundly affect the phenotype of antigen presenting cells (macrophages and dendritic cells), reducing their surface expression of co-stimulatory receptors, secretion of pro-inflammatory cytokines, and ability to present antigen to T cells (**3**). In terms of T cell responses, IVIg causes effector T cell apoptosis, suppression of pro-inflammatory cytokine secretion and skewing towards immunomodulatory T-regulatory cell responses required for establishment of early blastocyst tolerance (**4**,**5**). Finally, IVIg has also been shown to neutralize pathogenic auto-antibodies, but the relevance of this mechanism in human reproduction is unclear (**6**)^7^.

Our dosing scheme based upon the hypothesis that IVIg acts to reduce endometrial inflammation and improves endometrial or systemic tolerance to the implanting embryo (Fig. 2). By administering IVIg 5–10 days prior to embryo transfer at moderate doses (0.6–0.8 g/kg) we enable sufficient time for IVIg to prime antigen presenting cells towards tolerance, potentially increasing T regulatory cell numbers and enhancing their suppressive capacity^38,39^. It is unclear if lower IVIg doses (0.2–0.4 g/kg) have similar effects and high dose IVIg (1 g/kg) may suppress the normal inflammatory events required for implantation.

We were able to show that in both primary and secondary RIF, patients with severe RIF phenotypes ($\ge$ 5 failed high quality blastocyst transfers) seemed to benefit from IVIg (Table 3). Patients with severe RIF may be more likely to have a diagnosis of immune-mediated reproductive failure, whereas patients with moderate phenotypes may have other factors explaining RIF (such as embryo aneuploidy for example). From this data, we can estimate a sample size required for an RCT in patients with $\ge$ 5 failed ET. Expecting a LBR of 20% in the control group and averaging expected LBR with IVIg to be 60%; with α = 0.05 and β = 0.2, 23 patients per group are necessary for 80% power. There is a non-statistically significant trend towards increased live births in patients with moderate RIF (Table 3), but the cost–benefit of IVIg treatment in this group may not be justified. Indeed, estimating LBR to be 60% with IVIg and 40% without, 194 patients (97 per group) would be needed to determine if IVIg improves LBR in this patient population; our study is underpowered. However, even if we were able to show that IVIg is effective in improving LBR in patients with moderate RIF, the number needed to treat would be too high to ensure a cost/resource-effective treatment.

Another important finding from this cohort is that IVIg has a similar beneficial effect in both primary and secondary RIF. In a future RCT, both patient populations could be combined to facilitate recruitment. In our study, the miscarriage rate in the IVIg treated group (6.12%) was similar to that of the control group (7.49%) and lower than expected in the general population^40^. While IVIg has been shown to improve live birth rates in patients with higher order miscarriages^41,42^ our RIF patient population is not expected to be at higher risk of miscarriage^43^ and we do not expect IVIg to further reduce the risk of pregnancy loss compared to controls.

The main limitation of our study is its retrospective nature that might hold undetected biases. The control cohort was unmatched and had a lower number of previous transfers failures, possibly representing a better prognosis group than the IVIg treated group. However, that might emphasize the positive effect of IVIg in patients with poorer prognosis. Another limitation in this study is that the rate of PGT-A tested embryos is low. On one hand, knowing the ploidy of embryos before transfer, especially in women with more advanced maternal age, would have strengthened the diagnosis of ‘unexplained’ RIF. On the other hand, PGT-A is controversial in good-prognosis patients^44^, and may decrease the pregnancy rate per IVF cycle started^45^. We thus did not insist on PGT-A testing prior to IVIg treatment, especially in young patients with morphologically high-quality embryos. While the sample size is small, looking at both RIF groups combined, we have a sufficient cohort size for meaningful results. The number of patients with severe RIF is low compared to patients with moderate RIF, but we were still able to observe a beneficial effect of IVIg even after adjusting for maternal age. Finally, we recorded maternal age at embryo transfer and not maternal age at oocyte collection. In any subsequent study, both should be recorded to better assess association between IVIg outcomes and maternal age. Of note, the results of this study are not generalizable to all patients with RIF. We believe that only a subset of patients with severe RIF may benefit from IVIg.

Despite these promising results, it is too early to recommend routine administration of IVIg for RIF patients. IVIg is safe during pregnancy^15,16^, but a minority of patients will experience moderate to severe headache or infusion reaction post treatment; risks of anaphylaxis, aseptic meningitis, hemolytic anemia and blood borne pathogen transmission are possible, albeit extremely rare^46^. Furthermore, IVIg is used to treat a wide variety of severe medical conditions. It is a fractionated plasma product, one dose of IVIg is typically produced from over 1000 donors; one gram of IVIg can cost over 100 dollars (representing up to 5000$/infusion/patient) (pricing estimates from Hema-Quebec 2021–2023). During the recent COVID pandemic, a nation-wide blood product shortage prompted governing agencies to severely restrict access to IVIg, forcing medical practitioners to review their prescribing practices. Indeed, from January 2021 to December 31 2022, IVIg was unavailable for patients with RIF in Quebec. New internal guidelines were drafted in the province (Optimal usage of intravenous or subcutaneous immunoglobulins in fertility, cardiology and for other indications, INESS, October 2022) to protect from over-prescription of IVIg for patients with reproductive failure, with IVIg becoming available again only for patients with severe unexplained RIF as of January 2023. Indeed, during this time, we reviewed our own protocol for IVIg, now administering one dose 5–10 days prior to embryo transfer only repeating monthly dosages in patients with previous miscarriages. This is based on the thought that for RIF, immune modulation is needed only during the implantation period. The immunomodulatory effect of IVIg can last for up to 3 months post-infusion^47^, after which systemic maternal tolerance should already be established to the fetus^48^. By restricting IVIg access and dosage, we are contributing to blood product stewardship, ensuring only select patients receive IVIg rather than all patients with unexplained RIF.

## Conclusion

Patients with severe, unexplained RIF may benefit from IVIg. While a well-designed RCT is needed to confirm these results, clinical phenotype is important to consider. Indeed, patients with severe RIF may be more likely to have an underlying diagnosis of immune-mediated RIF. Repeating an RCT without proper patient selection would likely contribute to perpetuating clinical equipoise on IVIg use for reproductive failure. Furthermore, IVIg continues to be used as an off-label treatment for patients with RIF with very little regulation and high cost to the patient. Until RCT data confirms our results, IVIg should not be used for RIF outside of a research setting. IVIg-treated patients must be carefully monitored and tracked, ideally by including them in registries or cohort studies. This permits periodic practice audits for continued efficacy as well surveillance for IVIg side effects and adverse maternal, obstetrical, and neonatal outcomes. While IVIg is considered safe during pregnancy, off-label utilization should incorporate mechanisms to monitor ongoing patient safety. As such, patients should be screened for anemia, renal insufficiency and elevated liver enzymes for up to 3 months post IVIg as well as followed prospectively to record pregnancy and neonatal outcomes. As we continue to refine IVIg eligibility criteria, it is also important to seize the opportunity to better characterize the patient population that benefits from IVIg. Indeed, understanding the underlying immune mechanisms of RIF, developing a molecular diagnosis for IM-RIF and comprehending the potential mechanisms of action of IVIg will further enable targeted therapy.

### Supplementary Information

Supplementary Table 1.

## Data Availability

The datasets used and/or analysed during the current study are available from the corresponding author on reasonable request.

## Abbreviations

AMH: Anti-mullerian hormone
BMI: Body mass index
CARTR: Canadian assisted reproductive technologies register
ET: Embryo transfer
GW: Gestational week
IM-RIF: Immune mediated recurrent implantation failure
IVIg: Intravenous immunoglobulin
LBR: Live birth rate
LMWH: Low molecular weight heparin
MUHC: McGill University Health Center
MRIC: McGill University Health Center Reproductive Immunology Clinic
NK cell: Natural killer cell
PCOS: Polycystic ovarian syndrome
PGT-A: Pre-implantation genetic screening
RCT: Randomized controlled trial
RIF: Recurrent implantation failure
RIF-1: Primary recurrent implantation failure
RIF-2: Secondary recurrent implantation failure
uRIF: Unexplained recurrent implantation failure
