# Supplementary material for: Intravenous immunoglobulin for patients with unexplained recurrent implantation failure: a 6-year single center retrospective review of clinical outcomes
Source: Sci Rep. 2024 Feb 16;14:3876. doi: 10.1038/s41598-024-54423-z (PMC10873418; doi:10.1038/s41598-024-54423-z)
Supplement: Supplementary file 1 — Supplementary Table 1. [file 41598_2024_54423_MOESM1_ESM.docx]

|  | Primary RIF |  | Secondary RIF |  | p-value for interaction* | OR (combined)- no interaction | p-value (combined)-no interaction |
| --- | --- | --- | --- | --- | --- | --- | --- |
| Model | OR (95 %CI) | p-value | OR (95 %CI) |  |  |  |  |
| Crude | 5,60 (1.88-18.02) | 0.0026 | 3,21 (1.19-9.45) |  | 0.47 | 3,64 (1.78-7,67) | 0,0004 |
| Adjusted for age | 6,78 (2.13-24.35) | 0.0018 | 2,92 (1.06-8.69) | 0.043 | 0.47 | 3,50 (1.69-7.43) | 0.00084 |
| Adjusted for #failed embryo transfers | 8,35 (2,14-38.88) | 0.0036 | 3,22 (1,18-9.58) | 0.026 | 0.41 | 3,77 (1.77-8.33) | 0.0007 |
| Adjusted for age and #failed embryo transfers | 10,14 (2.44-52.13) | 0.0026 | 2.94 (1.06-8.82) | 0.043 | 0.41 | 3,63 (1.69-8.09) | 0.0011 |

**Supplemental appendix:**

**Table 1: Logistic regression analysis for association of IVIg with live birth with different adjustment models (control group used as reference)**

p-value for interaction between IVIg and type of RIF (primary vs secondary) obtained from a interaction factor (IVIg*type of RIF)

Age and number of previously failed embryo transfers as continuous variables
